# *Lactobacillus casei* UT1 Isolated from Northwest of Iran Traditional Curd Exerts Anti-proliferative and Apoptosis Inducing Effects in Human Colorectal Tumor HCT 116 Cells

**DOI:** 10.15171/apb.2020.016

**Published:** 2019-12-11

**Authors:** Mitra Rabiei, Gholamreza Zarrini, Majid Mahdavi

**Affiliations:** Department of Biology, Faculty of Natural Science, University of Tabriz, Tabriz, Iran.

**Keywords:** Apoptosis, HCT116 cells, *Lactobacillus*, Probiotic

## Abstract

***Purpose:*** The present study was mainly designed to assess anti-cancer effects of lactobacilli isolated from traditional dairy products, on HCT116 colorectal cancer cell lines.

***Methods:*** Traditional dairy products samples were collected from the region of Azarbayjan and the suspensions were cultured in MRS agar medium. The isolates were identified by biochemical and molecular methods. Isolated bacteria were cultured in MRS broth. Supernatants of the isolates cultures were collected and their cytotoxicity was evaluated on HCT116 cancer cells. Morphological changes of the treated cells by supernatant were observed using an inverted microscope. Cell metabolic activity was assessed by MTT assay. The morphology of apoptotic cells was examined using a fluorescent microscope. In cell cycle analysis, content measurement of DNA was performed by flow cytometry.

***Results:*** Out of 30 lactobacilli were isolated from dairy products samples, six isolates belong to curd samples. Cell-based assays showed that culture supernatant of one isolate (UT1) had a significant anticancer effect on colorectal HCT116 cell lines (*P*<0.05). The 16S rRNA sequence analysis revealed that the isolate UT1 was 99% compatible with Lactobacillus casei.

***Conclusion:*** It is noteworthy that the supernatant of L. casei UT1 can be candidate for studies on compounds having anti-cancer effect.

## Introduction

One of the most important emerging fields in the food industry is the concept of “food function” or “functional foods”. For individual’s health promotion and/or disease prevention, a word “food function” can be often used with the meaning of pharmacological effect of food-stuffs as well as their ingredients.^1^ Today, many of the dietary agents and natural health products have attracted the attention of scientists. One of them is probiotics.^2^ Within the functional foods, the use of probiotics is rapidly expanding.^3^ Probiotics are defined as “live microorganisms” that, when ad­ministered in adequate amounts, confer health benefits on the host.^4-8^ Probiotics can be bacteria, moulds, yeast. But most probiotics are bacteria. Among bacteria, lactic acid bacteria (LAB) are more popular, and most probiotics belong to this group.^3^ Probiotics, in the form of dairy foods containing LAB, have been consumed for centuries by humans.^9^

During the past decade, the importance of LAB in the maintenance of gut health has increasingly been recognized. Accordingly, the possible health-related benefits associated with consumption of LAB as a dietary supplement are well-documented. In particular, the potentiality of dietary LAB to prevent chronic diseases such as cancer is so promising. Researches indicate that fermented milk products have been shown to have strong chemopreventive activity towards carcinogenesis.^10^ A number of clinical studies have been performed on the ability of probiotic in the prevention, control and treatment of various cancers, especially the gastrointestinal tract. Due to the large quantities of probiotic bacteria in the gut, probiotics seem to be one of the most interesting candidates for the treatment of colorectal cancer.^11^ HCT116 cell line as a type of carcinoma, expresses transforming growth factor beta 1 and beta 2. In addition, HCT116 has a mutation in codon 13 of Ras proto-oncogene.^12^

In terms of useful properties, especially anti-cancer effects, the most important and common genera LAB in fermented foods, particularly dairy products, are lactobacilli.^13^ Some studies show that increase in lactobacilli in the gut, by competing with pathogenic bacteria, reduce the production of some mutagenic compounds.^9^ Some strains of lactobacilli and their fermented products could reduce the risks of certain types of cancer and inhibit the growth of certain tumors in vitro assays, animal studies, human studies, epidemiological and intervention studies.^14^

During this project, due to the major role of dairy products probiotics to control intestinal health, anti-cancer effects of lactobacilli isolated from traditional dairy products, on HCT116 colorectal cancer cell lines are examined.

## Materials and Methods

### Sampling and isolation

Traditional dairy products samples were taken aseptically and transferred to microbiology lab. Different dilutions of the samples were made in physiological saline and inoculated on MRS (De Man, Rogosa and Sharpe) agar (HiMedia, India). Inoculated plates were incubated anaerobically for 48 hours at 37˚C. Appeared colonies were examined by gram staining and catalase test and lactobacilli were isolated.^15^

### Strains identification

#### Morphological and biochemical identification

The isolates were identified following morphological and biochemical characterization according to Bergey’s Manual of Systematic Bacteriology, and Voges-Proskauer (VP), carbohydrate fermentation (glucose, galactose, lactose, arabinose, maltose, mannitol, sorbitol, sucrose, xylose, melibiose, raffinose, trehalose), arginine hydrolysis and growth at different temperatures (15˚C and 45˚C) tests were performed.^16-18^

#### Molecular identification

Molecular methods are important for bacterial identification.^19^ For molecular characterization, PCR analysis of 16S rRNA gene was performed using the universal primers and followed by agarose gel electrophoresis. Sequence (performed by Bioneer, Germany) homologies were examined by comparing the obtained sequence with those in the DNA databases (http//www.ncbi.nim.nih.gov/BLAST)^16,20-22^ and dendrogram of the isolates were depicted by Mega 5 software.

### Preparation of cell-free supernatant

All the isolated bacteria were cultured in MRS broth (Biolife, Italy) and were incubated for 24 hours at 37˚C. Optical density (OD) of cultures was measured at 600 nm and the number of bacteria was calculated (CFU [colony-forming unit]= OD × 8 × 10^8^). Supernatants were isolated by centrifugation (3000 rpm, 30 min) and were sterilized using a 0.22 µm filter.^23^

### Cell analysis

#### Cell culture

The HCT116 cell line were purchased from Pasteur institute of Iran and were cultured in RPMI (Gibco, England) medium with 10% FBS (Sigma, Germany).^24^

#### Cell viability assay

The cells were cultured in 96 wells plate. 0.5, 1, 1.5, 2 and 5 × 10^7^CFU/mL concentrations of the supernatants were added to the wells, respectively and cultures were incubated for 24, 48 and 72 hours at 37˚C and 5% CO_2_. Morphological changes of treated cells by supernatant were observedusing a microscope. After 72 h incubation, 20 μL of MTT solution (5 mg/ml) was added to each well and incubated for another 3 h. The medium was then removed and the blue formazan crystals were solubilized with 200 μL of Dimethyl sulfoxide (DMSO). MTT converted to formazan by metabolically viable cells and its absorbance was measured using an ELISA reader at 570 nm.^11,24,25^

#### Fluorescent staining

After cell culture and treatment for 72 h, 20 μL of trypsin was add­ed into each well. When cells had sloughed off, suspensions were transferred to micro-tubes. Dual fluorescent staining solutions (1 μL) containing 100 μg/mL acridine orange (AO) and 100 μg/mL ethidium bromide (EB) were added to each suspension and then covered with a coverslip.^26^ The morphology of apop­totic cells was examined using a fluorescent microscope.

#### Cell cycle assay

Primary steps were performed according to the fluorescence test. After transferring of cell suspensions to micro-tubes, the cells were washed twice with phosphate buffered solutions (PBS) and were centrifuged. The pellet was dissolved in 50 μL of cold PBS and 450 μL of cold ethanol for 1 hour at 4˚C. The cells were centrifuged again for 5 minutes and the pellet was washed with PBS and cells were centrifuged. Then, the pellet was dissolved in PBS containing RNAase enzyme (20 μg/mL) and the cells were incubated for 30 minutes in 37˚C. Then, the cells were incubated with propidium iodide (50 μg/mL) for 1 hour. After these steps, the result was examined by flow cytometry.^27^

#### Statistical analysis

All values are expressed as mean ± SD from three independent experiments. Statistical analysis was done using GraphPad Prism with two-way analysis of variance (ANOVA) and Tukey multiple comparison tests. *P*< 0.05 was considered statistically significant

## Results and Discussion

Typically isolated colonies of *Lactobacillus* appeared on MRS agar were circular, convex, opaque, smooth and white to creamy.^21,28^ During this study, 30 lactobacilli were isolated from dairy products samples and were designated as CT1 until CT3 (from “Lighvan” traditional cheese), JT1 until JT14 (from “jug” traditional cheese), ST1 until ST3 (from “Shoor” traditional dairy product), UT_1_ until UT_6_ (from traditional curd) and YT1 until YT2 (from traditional yogurt) based on their morphological characteristics. All isolates were Gram positive and catalase negative. Microscopic analyze of the isolates supernatants for primary screening identification of the highest strain in terms of cytotoxic activity was investigated (The results not shown) and the strain with the most cytotoxic effects (UT1) on tumor cells was selected for more evaluations.

**Figure 1:**
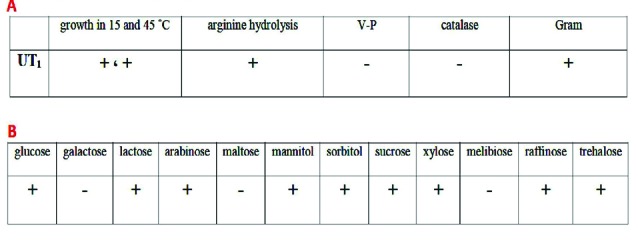
A: Biochemical characteristics of *Lactobacillus* sp. UT1. B: carbohydrate fermentation results of *Lactobacillus* sp. UT1. (+: growth, -: no growth)

**Figure 2:**
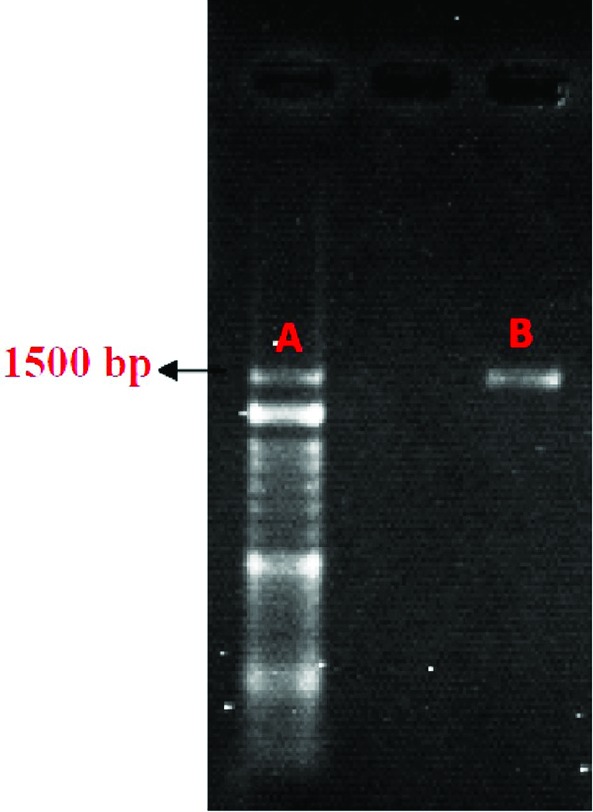
Agarose gel electrophoresis of PCR reaction product (A: DNA ladder: 50 bp, B: bound of UT1 sample, about 1500 bp).

**Figure 3:**
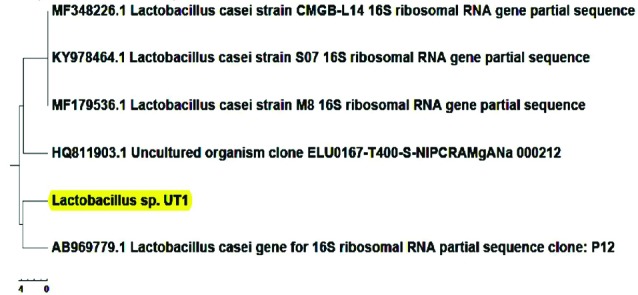
Dendogram of Lactobacillus sp. UT1. The distance between the reference strains of similar lactobacilli and isolated strain have been shown.

The biochemical characteristics of *Lactobacillus* sp. UT_1_ is shown in Figure 1. Agarose gel electrophoresis confirmed sequence amplification in the PCR reaction (Figure 2). According to the results, Sequence comparison using BLAST nucleotide database from the National Center for Biotechnology Information (NCBI) confirmed that isolated strain UT_1_ belonged to species of *Lactobacillus* was identified as *Lactobacillus casei* (Figure 3). The sequence of strain UT_1_ was 99% similar to that in the Gene bank and was registered with accession number MF506844 in NCBI.

**Figure 4:**
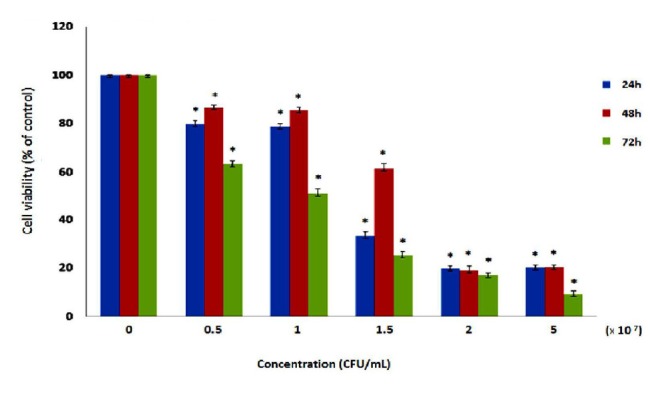
Effect of lactobacilli supernatant on HCT116 cells proliferation for 24, 48 and 72 hours. The inhibition of cancer cells proliferation increased in a dose and time dependent manner (* *P* < 0.05).

**Figure 5:**
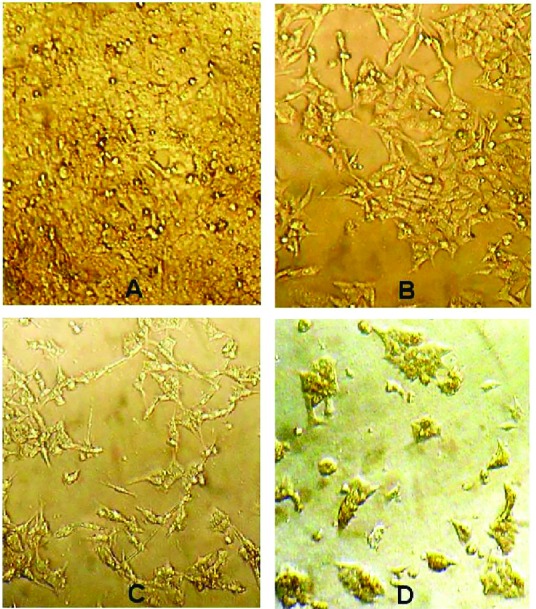
Morphological changes of HCT 116 cells affected by the IC_50_ value (1×10^7^ CFU/mL) of the bacterial supernatant (A: control cells, B: treated cells after 24 h, C: treated cells after 48 h, D: treated cells after 72 h).

**Figure 6:**
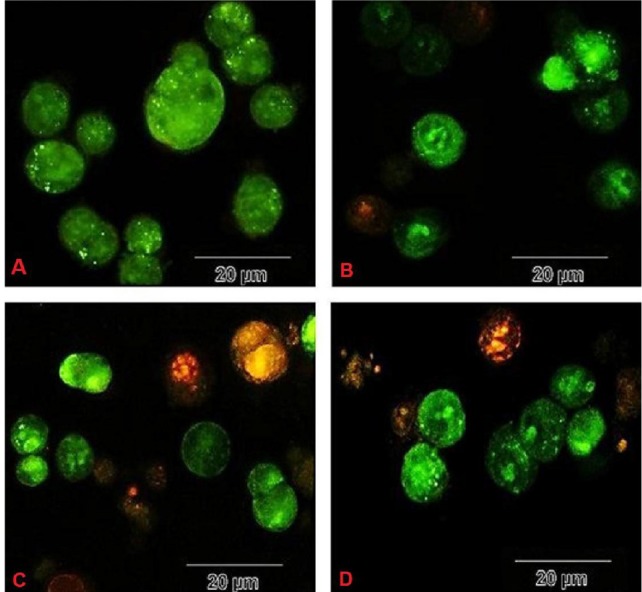
The incidence of apoptosis in HCT116 cells during treatment with metabolites from the strain UT1 (A: negative control cells, B: treated cells in 24h, C: treated cells in 48h, D: treated cells in 72h). The circular nucleus uniformly distributed in the center of the cell in untreated (control) cells (A). Nucleus showed yellow-green fluorescence by acridine orange (AO) staining and concentrated into a crescent or granular that located in 1 side of cells in early apoptotic cells (treated cells). After 48-72 h treatment, late apoptosis (orange spots) are seen (C and D).

**Figure 7:**
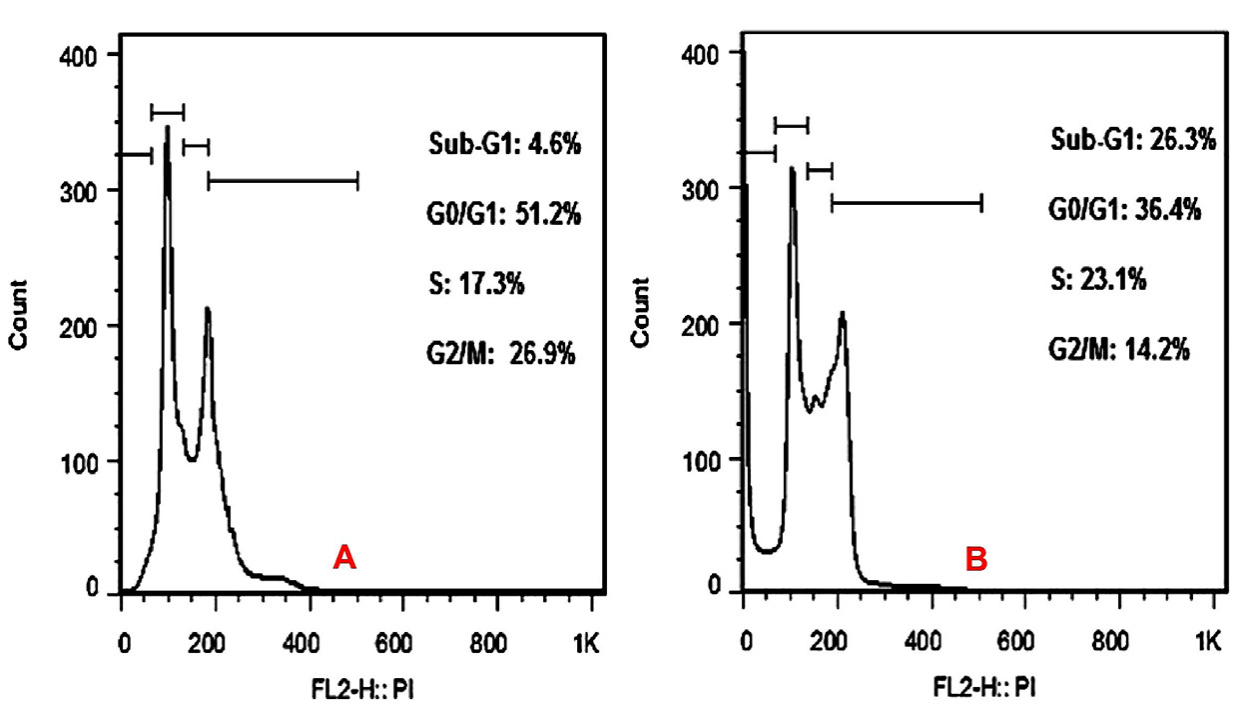
The results of cell cycle assay. Increasing the amount of cells in the sub G1 phase compared to control cells is seen. According to the results, the presence of cells in the sub G1 phase has increased from 4.6% in control (A) to 26.3% in the treatment for 72h (B).

Cell viability analysis using MTT assay provides evidence that traditional crude from UT1 has a cytotoxic activity (Figure 4). HCT 116 cells were incubated with MTT solution after treatment with 0.5-5×10^7^ CFU/mL of the bacterial supernatant. As shown in Figure 4, the inhibition of cancer cells proliferation increased in a dose and time-dependent manner. Following 72 h exposure, the IC_50_ value of the bacterial supernatant was calculated 1×10^7^ CFU/mL (Figure 4). As shown in inverted microscopy images (Figure 5), the untreated cells, apparently got wrinkled after treatment with an IC_50_ value of the bacterial supernatant. Cell rupturing and fragmentation observed as time increased. According to the result of fluorescent staining, after 24, 48 and 72 hours of the treatment, the incidence of apoptosis was observed (Figure 6). Apoptosis occurrence was also confirmed by flow cytometry in the cells. As shown in Figure 7, during treatment, the amount of cells in the sub G1 phase has increased compared to control cells. Increasing the presence of cells in the sub G1 phase indicated that the cells have transferred to the apoptotic stage. Therefore, evaluated metabolite in the present study can induce apoptosis in the HCT116 cells.

The results indicated that *L.casei* UT1 shows the inhibitory effect of cancer cells proliferation, as well as apoptosis induction activity. We are currently attempting to identify which components in the supernatant are responsible for the observed effects. The supernatant of strain seems to contain compounds with anti-proliferative effects. The inhibition of cancer cell growth by Lactobacillus has also been reported in other studies. Choi et al in their study in 2005 showed that *Lactobacillus acidophilus* extract decreases cell survival compared to controls 21%-28%. These results compared to the values obtained in the present study are much lower inhibitory effect. Soltan Dallal et al in 2015 examined the anticancer effect of supernatant of *L. acidophilus* on the Caco-2 cells at a concentration similar to this project. It was observed that cytotoxic effects were no more than 38%.^11^ Kahouli et al in 2015 evaluated cytotoxic effect of *Lactobacillus fermentum* supernatant on colorectal cancer cells.^25^ The results were almost identical to the results of this project. Er et al in 2015 examined anti-cancer effect of the large number lactobacilli on the Caco-2 cells.^30^ The results were not significant. Proper method and time for the release of metabolites can vindicate the significant cytotoxic effect of metabolites obtained from isolates in this project compared to other studies. In addition, the following resources to isolation of bacteria that are traditional dairy products, it is noteworthy that these compounds can significantly candidate for studies on compounds having anti-cancer effect.

## Conclusion

Results achieved in this study suggest that the use of lactobacilli probiotics can serve as a promising tool to prevent the incidence of colorectal cancer. The use of probiotics to reduce intestinal inflammatory responses that predispose to colorectal cancer reduces the risk of developing this disease. Laboratory studies suggest that probiotic bacteria prevent forming and proliferation of tumors. The new findings suggest that the use of lactobacilli can be a new method in the prevention of colorectal cancer.^11^ LAB pose as an avenue which can be exploited with both health and economic benefits.^31^ Further research is currently underway to investigate the anticancer properties and anticancer mechanism of lactobacilli.^14,24,32^ As well as, the identified isolate can be considered as starter cultures for their commercial uses.

## Ethical Issues

The current article does not contain any studies with human or animal subjects.

## Conflicts of interest

The authors declare that there is no conflict of interest.

## Acknowledgements

The authors appreciate the support of this investigation by the research council of University of Tabriz, Tabriz, Iran.
